# Zinc Oxide Nanoparticles—Solution-Based Synthesis and Characterizations

**DOI:** 10.3390/nano13111795

**Published:** 2023-06-02

**Authors:** Khagendra P. Bhandari, Dhurba R. Sapkota, Manoj K. Jamarkattel, Quenton Stillion, Robert W. Collins

**Affiliations:** 1 Department of Physics and Astronomy, Ohio Northern University, Ada, OH 45810, USA; q-stillion@onu.edu; 2 Wright Center for Photovoltaics Innovation & Commercialization, University of Toledo, Toledo, OH 43606, USA; dhurbaraj.sapkota@rockets.utoledo.edu (D.R.S.); manoj.jamarkattel@rockets.utoledo.edu (M.K.J.); robert.collins@utoledo.edu (R.W.C.)

**Keywords:** znc oxide, nanoparticle, absorbance, absorption coefficient, bandgap, ellipsometry, crystal structure, surface morphology

## Abstract

Zinc oxide (ZnO) nanoparticles have shown great potential because of their versatile and promising applications in different fields, including solar cells. Various methods of synthesizing ZnO materials have been reported. In this work, controlled synthesis of ZnO nanoparticles was achieved via a simple, cost-effective, and facile synthetic method. Using transmittance spectra and film thickness of ZnO, the optical band gap energies were calculated. For as-synthesized and annealed ZnO films, the bandgap energies were found to be 3.40 eV and 3.30 eV, respectively. The nature of the optical transition indicates that the material is a direct bandgap semiconductor. Spectroscopic ellipsometry (SE) analysis was used to extract dielectric functions where the onset of optical absorption of ZnO was observed at lower photon energy due to annealing of the nanoparticle film. Similarly, X-ray diffraction (XRD) and scanning electron microscopy (SEM) data revealed that the material is pure and crystalline in nature, with the average crystallite size of ~9 nm.

## 1. Introduction

In materials science, the ZnO is a wide bandgap II–VI semiconductor compound, the chemical bonding of which is largely covalent but with a substantial contribution from ionic bonding [1]. The ZnO can crystallize in the wurtzite, zinc blende, and rock salt crystal structures. The compound semiconductor has a tetrahedral bonding configuration, where each anion is surrounded by four cations at the corners of a tetrahedron, and each cation is surrounded by four anions, corresponding to the sp^3^ covalent bonding [2]. The compound zinc oxide (ZnO) is one of the most functional materials with remarkable and unique physical and chemical properties, such as strong chemical stability [3,4,5], high electrochemical coupling coefficients [6,7], and a broad spectrum of radiation absorption [8], along with high photostability [9].

Among the diversity of materials and needs, the oxide semiconductor ZnO is of great interest and is one of the versatile materials in materials science. The research interest in ZnO is growing every year, as can be observed from the rapid growth in the number of publications about the materials [10]. The material has a non-toxic nature and is cost-effective in producing both small and large scales. ZnO is considered as a leading candidate material for the next generation of the electronics industry, and as a practical material for medical devices [11,12]. Zinc oxide has also been considered as a promising anode material for Li-ion batteries because of its high theoretical capacity, environmental friendliness, abundance, and economical nature [13,14]. ZnO is recognized as an n-type, multi-functional semiconductor material, with a direct bandgap energy of ~3.37 eV and exciton binding energy up to 60 eV, even at room temperature, which is even higher than that of GaN [2,15,16,17]. This exceptional characteristic of ZnO makes it a lucrative material for room-temperature ultraviolet lasing devices [18,19]. Other applications of ZnO include optoelectronic and spintronic devices [20], UV light emitters [21], varistors [22,23], ceramic positive temperature coefficient thermistors [24,25], transparent high-power electronics [26,27], surface acoustic wave guides [28], piezoelectric transducers [29], chemical and gas sensor [30], solar cells [31,32,33], and piezoelectric nanogenerators [34]. As a result of its high emission efficiency, it is an important material for solid-state lighting technology [35]. It also provides opportunities for the formation of effective new optical and magnetic devices, such as spin-polarized solar cells, spin light-emitting diodes, and magneto-optical switches [36].

Apart from bulk ZnO and thin films/epilayers [37], several nanostructured polymorphic shapes of ZnO are available, such as nanorods [38,39], nanowires [33,40], nanoflowers [41,42], and nanoparticles [43,44,45]. These nanostructures have proven to be important for optoelectronic devices because of their large surface-to-volume ratios [46,47]. These various structures achieved by the nanostructures of ZnO make these materials unique, with a diverse set of characteristics and prospective uses in a variety of nanotechnology disciplines. Several methods for the synthesis of ZnO nanostructures have been reported, such as microemulsion synthesis [48,49], sol-gel techniques [50,51,52], mechanochemical processing [53,54], spray pyrolysis and drying [55,56], thermal decomposition of organic precursor [57,58], RF plasma synthesis [59,60], supercritical water processing [61,62], self-assembling [63], hydrothermal processing [64,65,66], vapor transport process [67,68], microwave assisted synthesis [69,70], direct precipitation, and homogeneous precipitation [71,72].

Out of all these deposition methods, hydrothermal, precipitation, colloidal, and sol-gel belong to the main category of the liquid-phase method of synthesis of nanomaterials. These methods are important because of their convenient operation procedures, simple synthetic route, and controllable particle size distribution. Hydrothermal synthesis can be used to fabricate nanomaterials at all temperature ranges and is considered one of the best methods for high-temperature synthesis. Similarly, the precipitation method is used to generate a pure and homogeneous nanomaterial. Product separation is necessary after precipitation and constant product quality throughout the precipitation process is sometimes challenging.

In the chemical vapor deposition (CVD) method, solid material is deposited using vapors obtained from the chemical reaction of the materials occurring on or in the locality of a normally heated substrate’s surface [73]. The generated thin film has uniform thickness with low porosity, even on a substrate with a complicated shape. This method is important to fabricate selective deposition, even on patterned substrates, and provides a pure material with economical production. On the other hand, CVD requires an expensive high-vacuum deposition system, making this deposition method not appropriate for all users.

In this work, we investigate the structural, morphological, and optical properties of ZnO nanoparticles obtained from the solvothermal synthesis route. The solvothermal method helps to speed up the reaction among the reactants and to enhance the crystal growth, resulting in self-assembly of nanoparticles in the solution. The synthesis is completed using zinc acetate and potassium hydroxide in the presence of methanol (Section 2). ZnO nanoparticles obtained from this method have already been used in quantum dot solar cells [74,75]. However, the authors realize that a detailed characterization, including a spectroscopic ellipsometry study, is necessary to further investigate its applications. We found this method to be a cost-effective and facile method for the synthesis of ZnO nanoparticles. The structure, morphology, and optical properties of the nanoparticles were investigated using XRD, SEM, SE, and a UV/Vis spectrophotometer.

## 2. Materials and Methods

### 2.1. Materials

Zinc acetate (ZnAc_2_, 99.99%, Sigma Aldrich, St. Louis, MO, USA), potassium hydroxide (KOH, certified ACS pellets, Fisher Scientific, Waltham, MA, USA), chloroform (anhydrous, 99+%, Sigma Aldrich), and methanol (MeOH, anhydrous, 99.8%, Sigma Aldrich) were purchased and used as received.

### 2.2. Nanoparticle Synthesis

Synthesis of ZnO nanoparticles was conducted using a modified form of the solvothermal method described elsewhere [74,75,76]. Two solutions were prepared in separate containers: solution 1 was prepared by dissolving 0.55 g of zinc acetate (Zn(CH_3_COO)_2_) in 25 mL of methanol (MeOH). The solution was stirred for an hour at a hot plate temperature of 60 °C. Solution 2 was similarly prepared by dissolving 0.28 g of potassium hydroxide (KOH) in 12.5 mL of methanol by stirring at the same hotplate temperature as for solution 1. Solution 2 was added to solution 1 using a titration process with the help of a burette and the reaction was left to run for 2 h at 60 °C for the nucleation and growth of the nanoparticles. After 2 h, the solution mixture was centrifuged to extract nanoparticles and re-suspended in methanol. This process was repeated three times, and finally, dry ZnO nanoparticles were suspended in chloroform for characterization in solution and thin films. The synthesis of ZnO nanoparticles was summarized using the following balanced equation:

$$\mathrm{Zn}{\left({\mathrm{CH}}_{3}\mathrm{COO}\right)}_{2}+2\mathrm{KOH}\to \mathrm{Zn}{\left(\mathrm{OH}\right)}_{2}+2{\mathrm{CH}}_{3}\mathrm{COOK}\;\mathrm{Zn}{\left(\mathrm{OH}\right)}_{2}\;\to \;\mathrm{ZnO}+H_{2}O$$

### 2.3. Thin Film Fabrication

ZnO nanoparticles thin films were fabricated using the spin coating process. A ZnO nanoparticle solution of the desired concentration was prepared in chloroform. The obtained solution was spin-coated on the Fisher brand borosilicate glass and soda lime glass substrates at 1000 rpm for 10 s and 4000 rpm for 25 s at room temperature (23 °C). The prepared films were annealed at different hotplate temperatures for different times.

### 2.4. Characterization Methods

A field emission scanning electron microscope (Hitachi S-4800, Japan) was used to acquire SEM images of ZnO surface. The XRD patterns of the ZnO films were taken using a Rigaku Ultima III X-ray diffractometer (Rigaku, USA) fitted with a small-angle X-ray scattering at 40 kV accelerating voltage and 44 mA current. Dielectric functions were obtained using a spectroscopic ellipsometry study. To obtain SE data, a rotating-compensator ellipsometer J. A. Woollam Co., (St. Lincoln, NE, USA) M-2000 DI was used. Unpolarized absorbance and transmittance spectra of the films were taken using a Shimadzu UV 2401PC Spectrophotometer. Spectroscopic ellipsometry data were taken to identify their dielectric functions. It is important to note that all these measurements were conducted at room temperature.

## 3. Results and Discussions

### 3.1. Phase Analysis and Surface Morphology

**Figure 1:**
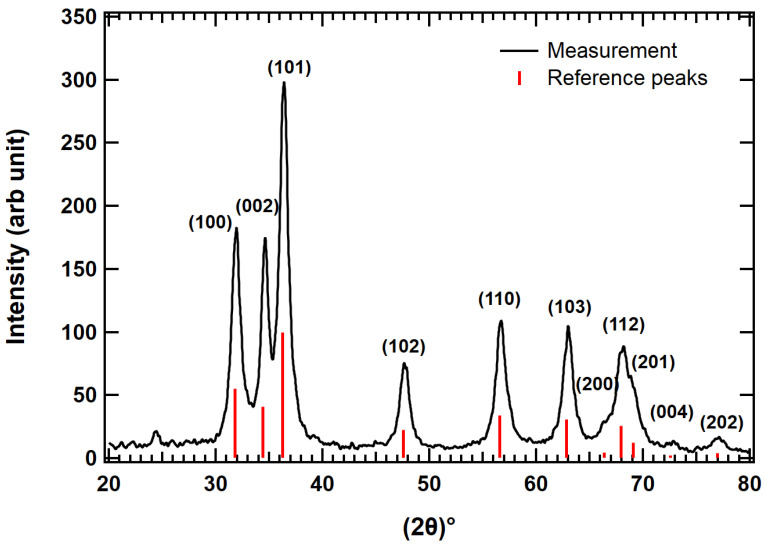
XRD patterns of annealed film of ZnO nanoparticles deposited on borosilicate glass substrate (Fisher brand microscope slide). The film was heated at 300 °C for 30 min.

To understand the purity and crystallinity of the ZnO thin films, XRD data were collected from as-synthesized film and an annealed film using Cu Kα radiation (*λ* = 1.54059 Å) in focused beam geometry. The XRD patterns obtained from the as-synthesized film are shown in Figure S1. Figure 1 shows XRD spectra of ZnO film annealed at 300 °C for 30 min. By comparing Figure 1 and Figure S1, it can be seen that the annealed film is more crystalline as compared to the as-synthesized film. All XRD peaks are indexed by the hexagonal wurtzite structure (#PDF 98-000-0483) of ZnO matching exactly with the standard peaks for the wurtzite phase represented by vertical lines, as shown in Figure 1. These standard reference peaks were obtained from MDI JADE (Jade^TM^ computer software from Materials Data Inc., St. Livermore, CA, USA). There is no evidence of traces of any other materials and sharp diffraction peaks are indicative of the good crystallinity and phase purity of the materials. The intense and sharp peaks of the XRD pattern indicate phase purity of ZnO nanoparticles. Further analysis of the material was done using Bragg’s law to identify the lattice constants, lattice spacing, and crystallite sizes.

The equation representing Bragg’s law of diffraction [77] is

(1) $$2d_{hkl}sin\theta =n\lambda$$

where n is the order of diffraction, λ is X-ray wavelength, and $d_{hkl}$ is the lattice interplanar spacing. For hexagonal structure, the plane spacing $d_{hkl}$ is related to the lattice constants a=b, c and the Miller indices (h, k, l) by the relation [77]:

(2) $$\frac{1}{d_{\left(hkl\right)}^{2}}=\frac{4}{3}\left(\frac{h^{2}+hk+k^{2}}{a^{2}}\right)+\frac{l^{2}}{c^{2}}$$

Using Bragg’s law for *n* = 1, Equation (3) reduces to

(3) $$sin^{2}\theta =\frac{\lambda^{2}}{4a^{2}}\left[\frac{4}{3}\left(h^{2}+hk+k^{2}\right)+{\left(\frac{a}{c}\right)}^{2}l^{2}\right]$$

The above equation for ⟨100⟩ and ⟨002⟩ planes, for example, becomes $a=\frac{\lambda }{\sqrt{3}sin\theta }$ and $c=\frac{\lambda }{sin\theta }$. Using *λ* = 1.54059 Å and 2θ for two planes 31.767° and 34.419°, lattice constants were calculated as a=b=3.2499 Å and c=5.2070 Å, respectively. These values match very well with the previous work calculated by Bindu et al. [78]. The interplanar spacing $d_{hkl}$ were calculated using Bragg’s law from XRD patterns for some major XRD peaks corresponding to ⟨hkl⟩ planes and were compared with reference data, as shown in Table 1.

The crystallite size (*D*) was also calculated in accordance with the Debye–Scherrer formula [44,79]:

(4) $$D=\frac{k\lambda }{\beta cos\theta }$$

In the above formula, k is a dimensionless shape factor (~0.90), λ is the wavelength of the incident X-ray, *D* is the crystallite size, θ is the Bragg’s angle, and β is the full width at half maxima (FWHM) of the corresponding peak. The *β* values were calculated using IGOR-Pro 9 Scientific data analysis software by Wave Metrics. The crystallite size was rounded to closest whole number in nanometer, as shown in Table 1. The average crystallite size for synthesized ZnO nanoparticle is 9 ± 2 nm. The average crystallite size calculated in this work is one of the smallest sizes obtained for ZnO nanoparticles [80,81,82]. The crystallite size is assumed to be the size of the smallest crystal, whereas the particle size indicates size of nanoparticle.

**Figure 2:**
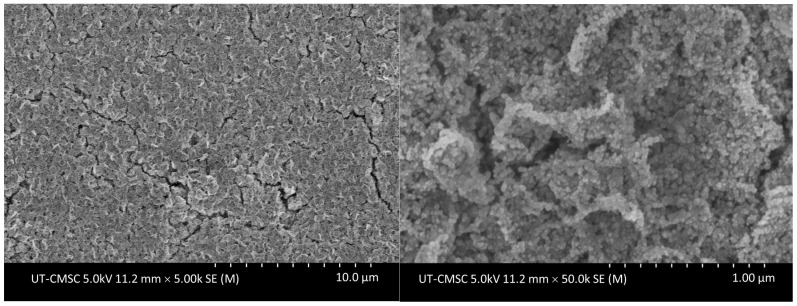
Scanning electron microscopy (SEM) images of ZnO nanoparticles. The nanoparticle thin film was spin-coated and SEM images were taken without the prior heat treatment.

Figure 2 shows SEM results of as-synthesized ZnO nanoparticles deposited on glass substrate at two different magnifications. As shown in Figure 2, these nanoparticles have very small sizes with no clear shapes but in terms of sizes they are larger than their crystallite sizes estimated by the Debye–Scherrer equation, as nanoparticles are an agglomerated form of crystallite. All these images were taken at 5.0 kV accelerating voltage using a lower SE detector to minimize charging resulting from the low carrier concentration of undoped nanoparticles.

ZnO nanoparticles can have different shapes and sizes based on the adoption of precursors and experimental procedures. The formation of nanoparticles usually takes place through the nucleation and growth processes. According to the Gibbs–Curies–Wulff theorem, the shape of a crystal depends on the surface free energy of individual crystallographic faces and the final shape of the nanoparticles is determined in such a way that the total free energy of the system is minimized [83]. In general, spherical nanoparticles are expected as the spherical shape, has the minimum surface energy for a given volume. By adjusting the reaction time, amount of precursors, temperature and pH, and the type of material used for the synthesis, nanostructures with a controlled size and shape, such as nanorods, nanowires, nanobelts, and nanostars, can be synthesized [84,85].

### 3.2. Optical Properties Using Spectroscopic Ellipsometry

Through careful analysis of XRD and SEM results, we see that ZnO nanoparticles can be considered as a suitable material for photovoltaic (PV) applications. Another way of finding the suitability of these ZnO nanoparticles in PV applications is to study their optical properties using dielectric functions. Optical responses of nanocrystalline ZnO thin films, in the form of energy-dependent complex dielectric functions, $\epsilon \left(E\right)=\epsilon_{1}\left(E\right)+i\epsilon_{2}\left(E\right),$ enables findings of optical and morphological properties applicable to PV and other electronic devices. To find dielectric constants at room temperature, external spectroscopic ellipsometry (ex-situ SE) data were collected from a ZnO thin film deposited on soda lime glass using the spin coating method. The ZnO thin film was annealed at a hot plate temperature of 300 °C for 30 min before taking the SE data. The SE spectra were collected ex situ at a 70° angle of incidence using a M2000 SE system with a back-side tape collecting 635 data points over a spectral range from 0.734 to 4.00 eV.

The complex dielectric functions for the ZnO thin film were parameterized by using two critical point parabolic bands (CPPB) and one Tauc-Lorentz [86] oscillator, with the detailed procedure described elsewhere [87]. Similarly, the film surface roughness layer was modeled using a Bruggeman effective medium approximation (EMA) [88,89]. From the SE analysis, the surface roughness and bulk layer thickness of the nanoparticle thin film sample are $d_{s}$ = 57 ± 1 nm and $d_{b}$ = 339 ± 1 nm, respectively. Similarly, total effective thickness of the film and bandgap energy of the material are $d_{eff}$ = 380 ± 1 nm and 3.22 ± 0.01 eV, respectively.

**Figure 3:**
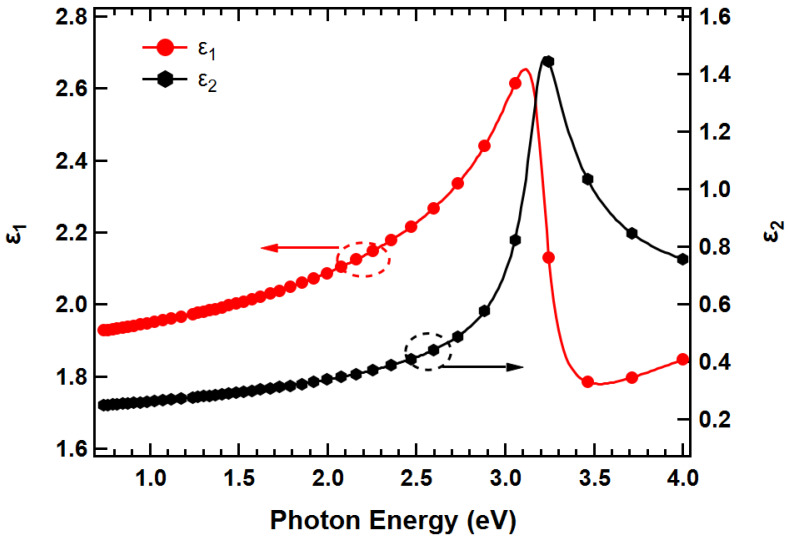
Dielectric function spectra for ZnO nanoparticle thin film measured by SE at room temperature. The red curve represents ε_1_ plotted to the left y-axis and is represented by an arrow with a circle and black curve represents ε_2_ as shown by a circle with an arrow to the right.

**Figure 4:**
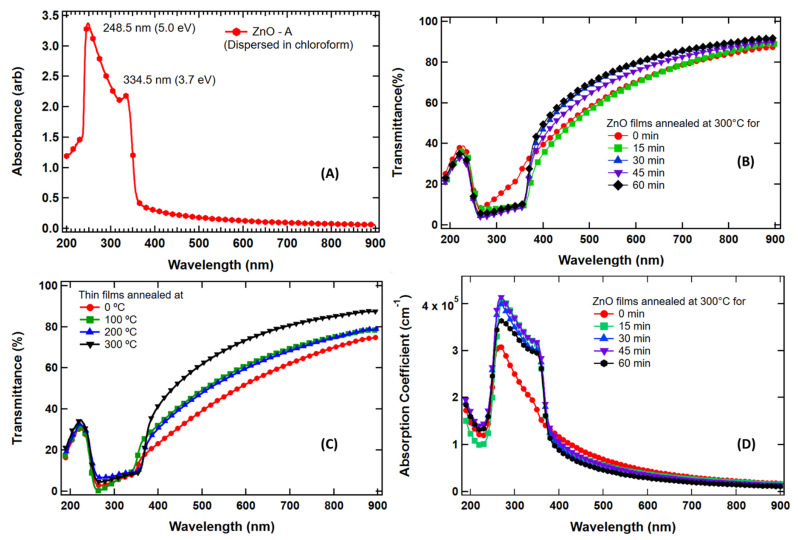
VU/Vis/NIR spectroscopy results: (**A**) Absorbance spectrum of as-obtained ZnO nanoparticles dispersed in chloroform. (**B**) Transmission % of ZnO thin film of ~100 nm before and after annealing the films. (**C**) Temperature-dependent transmission of ZnO thin films annealed for 30 min. When temperature is increased, transmission in infrared and visible regions is increased, whereas it is decreased in the band edge absorption regions. (**D**) Absorption coefficients (α, cm^−1^) of thin films of ZnO with respect to wavelength in nm. Small changes in α were observed when the films were annealed for different time intervals.

After finding thicknesses from the parametric models, numerical inversion [90] was used to extract dielectric constants at each spectral point for the sample over the measured spectral range. The complex dielectric response function $\epsilon \left(E\right)$ in the spectral range of 0.734 eV to 4.00 eV for the thin film of ZnO nanoparticles, as obtained from fitting the model, is shown in Figure 3. The dielectric function shown is comparable to the previously published work [91,92,93]. The imaginary part of dielectric function $\epsilon_{2}$ shows a characteristic peak at ~3.22 eV, with an onset of energy at ~3.0 eV for the nanoparticle film. The onset of absorption corresponds to the absorption edge of ZnO, a direct bandgap semiconductor, which matches well with the values calculated in Figure 4. The onset of energy found in this work is ~0.36 eV smaller than Girish Lakhwani et al. [91] for a similar ZnO nanoparticle film, but the value is similar to the single crystalline ZnO [92]. Even though the onset of absorption was expected to be higher for nanoparticle thin film due to the quantum confinement effect, it is smaller and similar to the bulk value due the annealing effect. The $\epsilon_{1}\left(E\right)$ and $\epsilon_{2}\left(E\right)$ spectra of the ZnO nanoparticle thin film calculated in this work are also slightly smaller in magnitude than that in the work of Girish Lakhwani et al. [91], as shown in Figure 3. At higher temperatures, nanoparticle thin films behave like a bulk material.

### 3.3. Unpolarized Absorbance and Transmittance

The optical absorbance spectrum of synthesized ZnO nanoparticles dispersed in chloroform was obtained using UV/Vis/NIR absorption spectroscopy, as shown in Figure 4A. The spectrum displays the first exciton absorption peak at a 334.5 nm (3.7 eV) wavelength, much below the bulk bandgap wavelength of 368 nm [2]. The first exciton absorption peak attributes the approximate intrinsic bandgap of ZnO due to the electron transitions from the valence band to the conduction band ($O_{2p}\to Zn_{3d}$) [94]. In addition, the sharp absorption at the band edge and narrow peak position reveal that the size distribution of the nanoparticles is small. The absorbance peak position (334.5 nm) obtained in this work lies at smaller wavelength than from previous research, where it was in the range of 355 nm to 380 nm [44,95,96,97,98,99]. The weak absorption area range includes the whole visible spectrum region (400 nm to 700 nm) and some part of the infrared spectrum (>700 nm) in the measurement range. After the first exciton peak, a monotonical increase in the absorption of light continues until the effect of glass at the middle ultraviolet (MUV) region breaks at 248.5 nm, as shown in Figure 4A. Another experiment was conducted where all KOH solution was mixed into the ZnAc_2_ solution simultaneously at once instead of via dropwise addition. The poly-dispersed ZnO nanoparticles obtained are shown in the Supplementary Information Figure S2.

At the absorbance edge, absorbance is contributed by the largest size nanoparticles, whereas at the region of absorbance maximum, absorbance contribution is from all particles. There must be a threshold value of wavelength where absorption just begins and is called threshold wavelength ($\lambda_{S}$). The threshold wavelength is 354.6 nm, only 20 nm higher than first exciton absorption peak, as shown in the Figure S3.

Transmittance % spectra of the ZnO nanoparticles of thin films of ~100 nm before and after annealing are shown in Figure 4B. In the Figure 4B, the red line represents transmission from as-synthesized film and other data represent transmission from annealed films. Five films of approximately similar thicknesses were prepared and four films were annealed at 300 °C for different times, as shown. The optimized annealing temperature of 300 °C was estimated based on the transmission measurement of four ZnO thin films at different temperatures, as shown in Figure 4C. When the film is annealed, traces of solvent and other byproducts evaporate, and defect states in the films are minimized or removed. The film becomes more crystalline which may be responsible for scattering the incident light and lowering the transmission. The small change in transmission in the visible range of the spectrum may be due to the slightly different thicknesses of the films. As shown in Figure 4B, in the visible range of spectrum, all films present a high average transmittance greater than 60%, indicating low absorbance and low reflectance, which confirms that these ZnO thin films can be used as antireflection coating in solar cells working mainly in the visible region, such as silicon solar cells [31,100]; in a large spectrum, such as GaN-based concentration solar cells [101]; or as a window layer in quantum dots solar cells [74].

The optical absorption measurement near the fundamental absorption edge is a standard method for the assessment of the bandgap energy. The variation of the optical absorption coefficient with incident wavelength (or energy) helps to explain the band structure and the type of transition of electrons. The optical absorption coefficient ($\alpha \left(\lambda \right)$) of the ZnO thin films were calculated using Beer–Lambert’s relation [102]:

(5) $$I=I_{0}e^{-\alpha \left(\lambda \right)x}\to \alpha \left(\lambda \right)=2.303\frac{A}{t}$$

where A represents the absorbance, and t represents film thickness. The basic procedure for absorption coefficient analysis is to obtain optical absorbance (*A*) data from transmittance of ZnO thin films taken from UV/Vis/NIR spectrophotometer by the relation: $A=2-log_{10}\left(T\%\right)$. As shown in Figure 4D, there is only a small change in optical absorption coefficient with respect to the temperature but at the band edge, the absorption coefficient is sharply increased with respect to as-synthesized film. This increase in α at the band edge may be attributed to the onset of interband transitions, which is more efficient in the case of annealed (crystalline) films than as-synthesized (less crystalline) ones. The absorption coefficient depends on the material’s extinction coefficient (κ: how strongly a material absorbs light of a particular wavelength) and the wavelength of light (λ) being absorbed by the relation: $\alpha =\frac{4\pi \kappa }{\lambda }$. The κ value depends on the type of bandgap (direct vs. indirect) and the λ depends on the bandgap of the material.

The optical bandgap of the ZnO is estimated using Tauc’s relation [103]:

(6) $$\alpha h\nu =A{\left(h\nu -E_{g}\right)}^{n}$$

where *A* is a constant, hν incident photon’s energy, and $E_{g}$ is the bandgap of the material to be determined. The factor n depends on the nature of the electron transition and is equal to ½ and 2 for the direct and indirect bandgap transitions, respectively.

**Figure 5:**
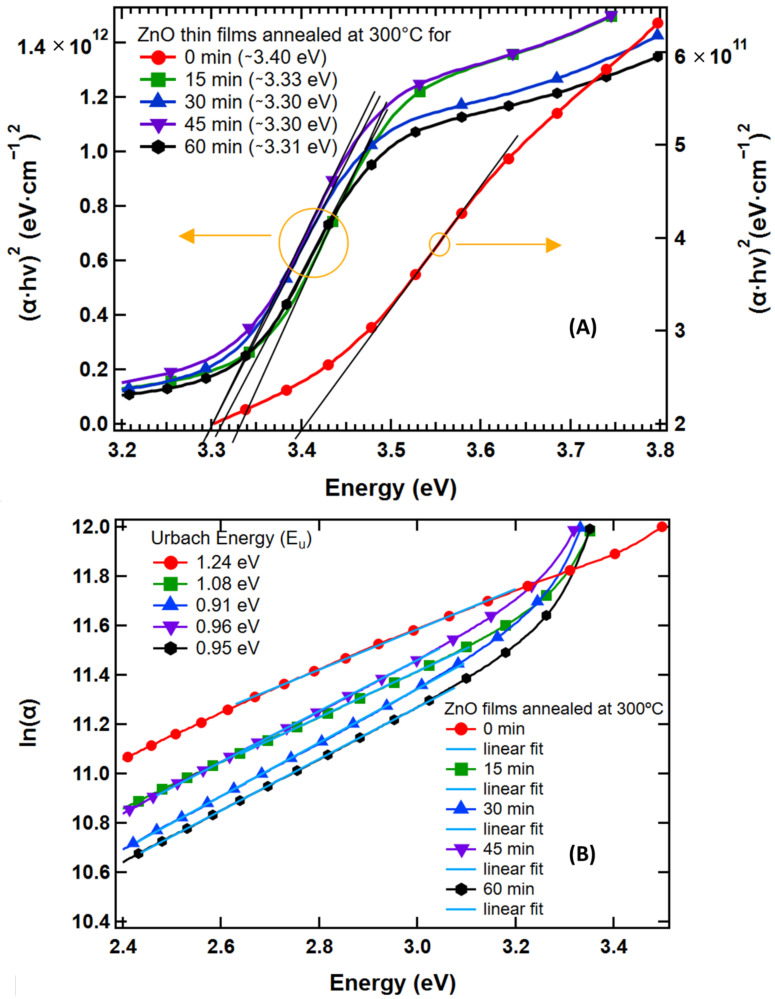
(**A**) Plot of ${\left(\alpha h\nu \right)}^{2}$ vs. hν of ZnO nanoparticles’ thin films. The red curve is from the as-synthesized film with *y*-axis to the right and the rest are from the heated films with *y*-axis to the left. Yellow circles and arrows represent directions of y-axes for respective curves. (**B**) Urbach energy calculation of ZnO nanoparticles. Low $E_{u}$ indicates that annealed films are pure & crystalline. More defect states are present in the as-synthesized film, as depicted by higher $E_{u}$.

Figure 5A gives the Tauc plot for ZnO, where α times the hν to the second power is plotted versus the hν. The second power of αhν is used as ZnO is well known to have a direct allowed transition. The characteristic features of Tauc plot are evident: at low photon energies, the absorption approaches insignificant—the material is transparent; near the bandgap value, the absorption gets stronger and shows a region of linearity in this squared-exponent plot. The linear fit applied at the linear region is extrapolated to the *x*-axis intercept to find the bandgap value. The direct bandgap values decreased from 3.40 eV at room temperature to 3.33 eV when the film was annealed for 15 min at 300 °C. The bandgap continued decreasing and saturated after 30 min at 3.30 eV. Temperature variation of the energy gap in semiconductors is known to be due to the effects of lattice dilation (expansion) and electron–phonon interaction or phonon-induced atomic vibrations [104,105,106]. Both experimental and theoretical studies conducted by Zhang et al. and others [107] have shown that bandgap decreases as temperature increases. The phenomenon is more prevalent in ionic compounds, such as ZnO, than covalent compounds due to stronger lattice expansion with temperature. The bandgap of the annealed sample matches very well with some of the previous results [99,108].

An obvious redshift in the absorption edge was observed for the ZnO film with respect to the exciton peak observed in the solution, as shown in Figure 4A. This might be due to the increased dielectric constant and substantial electronic coupling when the nanoparticles come close together after the evaporation of the solvent. In addition, in thin films, effects due to interference might occur that not only lead to unexpected absorbance values but also to peak shifts.

Figure 5A displays lower and upper deviations from the region of linear behavior. On the lower energy side, the deviation from the linear region can be associated with defect absorption states located near the band edge. This phenomenon was first investigated by Urbach [85] in silver halides, and in subsequent years, was applied to a large number of other crystals, therefore, it is known as an “Urbach Tail”. These defect absorption states exhibit an exponential energy dependence corresponding to a typical distribution of density of states. On the higher energy side, saturation of available density of states is responsible for a leveling out of absorbance strength [103].

Modification of band structure, which may be due to the introduction of defects in the material, can be recognized by measuring the material’s Urbach energy. It was found that the absorption coefficient near the band edge shows an exponential dependence on photon energy given by [109,110]

(7) $$\alpha \left(h\nu \right)=\alpha_{0}\exp\left(\frac{h\nu -E_{0}}{E_{U}}\right)$$

where $E_{0}$ and $\alpha_{0}$ are characteristic parameters of the material, and $E_{U}$, an inverse logarithmic slope of absorption coefficient, is the Urbach energy interpreted as the width of the tails of localized states, associated with the amorphous state, in the bandgap. To calculate the Urbach energy, a plot of lnα versus photon energy was made, as shown in Figure 5B. Then, the value of $E_{U}$ was calculated by taking the reciprocal of the slopes of the linear portion in the lower photon energy region of the curve. The minimum Urbach energy of 0.91 eV was found from a film heated at 300 °C for 30 min and maximum Urbach energy of 1.24 eV was found from the as-synthesized film. The higher $E_{U}$ from the as-synthesized film is due to the impure state of the material. Factors governing the Urbach energy are structural disorder, imperfection in stoichiometry and unpassivated surface state. Structural disorder may be higher in the as-synthesized nanoparticle thin film. Imperfection in stoichiometry may be caused by residual materials left after synthesis. In addition, dangling bonds disappear from the surface of the nanoparticles when the film is annealed.

## 4. Conclusions

In this work, we have reported the synthesis of monodispersed ZnO nanoparticles with a hexagonal wurtzite structure via the solvothermal synthesis method using ZnAc_2_ as zinc precursor and KOH as oxygen precursor. The nanoparticles were confirmed using XRD, SEM micrograph analysis, SP, and UV/Vis/NIR spectroscopy study. The SEM images showed identical morphology of ZnO nanoparticles with uniform particle size distribution. The XRD analyses proved the crystalline structure of the particles. The average crystallite size obtained from Scherrer’s equation was ~9 nm, one of the smallest for ZnO nanoparticles. Optical and morphological properties of ZnO nanoparticle film were also studied using ellipsometry in the spectral range from 0.734 eV to 4.00 eV. The measured spectra revealed the distinct band edge structures at 3.22 eV, ~0.1 eV smaller than found in another method. The sharp first exciton peak revealed that the ZnO nanoparticle size in these samples were nearly monodisperse. The first exciton peak in solution measurement dispersed in chloroform was at 334.5 nm (~3.7 eV), with threshold absorption wavelength of 354.6 nm (~3.5 eV). The first exciton peak was found red shifted in thin film measurement relative to nanoparticles in solution with a value of 350 nm (~3.5 eV). The red shift in the first exciton peak was due to the increased dielectric constant and substantial electronic coupling of nanoparticles. As expected, the bandgap energy transition of ZnO nanoparticles was found to be directly allowed, with values of 3.40 eV and 3.30 eV, respectively, for as-synthesized and annealed films. Our investigation revealed that the nanoparticles fabricated in this work have a wide range of applications, especially in the electro-technological industries, such as photoelectronics, field emitter, sensors, UV lasers, and solar cells.

## Figures and Tables

**Table 1:** Interplanar spacing from XRD, reference for corresponding ⟨hkl⟩ planes, FWHM, and crystallite size. The average crystallite size is ~9 nm.

⟨hkl⟩	$d_{hkl}\;Å$	$d_{Ref}\;Å$	$FWHM,\;\beta \;\left({}^{\circ}\right)$	$D\;\left(nm\right)$
⟨100⟩	2.8028	2.8146	1.003	8
⟨002⟩	2.5894	2.6035	0.714	12
⟨101⟩	2.4686	2.4760	0.793	11
⟨102⟩	1.9058	1.9112	0.935	9
⟨110⟩	1.6221	1.6250	1.041	9
⟨103⟩	1.4745	1.4774	0.981	10
⟨112⟩	1.3734	1.3785	1.632	6
⟨201⟩	1.2369	1.3598	1.290	8

## Data Availability

No new data were created.

## Supplementary Materials

The following supporting information can be downloaded at: https://www.mdpi.com/article/10.3390/nano13111795/s1, Figure S1: XRD patterns of as-synthesized ZnO thin film. The film was deposited by using a spin coating process. The material is less crystalline because the peak intensities are comparatively lower; Figure S2: Absorbance spectrum of ZnO nanoparticles dispersed in chloroform. Synthesis was done by mixing all KOH solution in methanol at once into all ZnAc_2_ solution in methanol; Figure S3: (*A*/*λ*)^2^ vs. 1/*λ* of ZnO nanoparticles dispersed in chloroform. References [111,112] are cited in the Supplementary Materials.

## References

[1] Patil L., Bari A., Shinde M., Deo V., Kaushik M. (2009). Zinc Oxide: Fundamentals Materials and Device Technology.

[2] Özgür Ü., Avrutin V., Morkoç H., Henini M. (2018). Chapter 16—Zinc Oxide Materials and Devices Grown by Molecular Beam Epitaxy. Molecular Beam Epitaxy.

[3] Olvera M., Maldonado A., Asomoza R. (2000). Chemical stability of doped ZnO thin films. J. Mater. Sci. Mater. Electron..

[4] Lee W., Yeop J., Heo J., Yoon Y.J., Park S.Y., Jeong J., Shin Y.S., Kim J.W., An N.G., Kim D.S. (2020). High colloidal stability ZnO nanoparticles independent on solvent polarity and their application in polymer solar cells. Sci. Rep..

[5] Heinonen S., Nikkanen J.-P., Huttunen-Saarivirta E., Levänen E. (2017). Investigation of long-term chemical stability of structured ZnO films in aqueous solutions of varying conditions. Thin Solid Film..

[6] Deschanvres J.L., Rey P., Delabouglise G., Labeau M., Joubert J.C., Peuzin J.C. (1992). Characterization of piezoelectric properties of zinc oxide thin films deposited on silicon for sensors applications. Sens. Actuators A Phys..

[7] Membetsi R.O.-N.Z.M., Gnanga H., Omanda H., Foucaran A. (2015). Microwave Analysis and Electrical Properties of ZnO thin Films Prepared by RF Magnetron Sputtering. Int. J. Sci. Res. IJSR.

[8] Segets D., Gradl J., Taylor R.K., Vassilev V., Peukert W. (2009). Analysis of Optical Absorbance Spectra for the Determination of ZnO Nanoparticle Size Distribution, Solubility, and Surface Energy. ACS Nano.

[9] Wahyuni E.T., Diantariani N.P., Kartini I., Kuncaka A. (2022). Enhancement of the photostability and visible photoactivity of ZnO photocatalyst used for reduction of Cr(VI) ions. Results Eng..

[10] Ayoub I., Kumar V., Abolhassani R., Sehgal R., Sharma V., Sehgal R., Swart H.C., Mishra Y.K. (2022). Advances in ZnO: Manipulation of defects for enhancing their technological potentials. Nanotechnol. Rev..

[11] Kumar V., Ntwaeaborwa O., Soga T., Dutta V., Swart H. (2017). Rare Earth Doped Zinc Oxide Nanophosphor Powder: A Future Material for Solid State Lighting and Solar Cells. ACS Photonics.

[12] Capper P., Kasap S.O., Willoughby A., Litton C.W., Reynolds D.C., Collins T.C. (2011). Zinc Oxide Materials for Electronic and Optoelectronic Device Applications.

[13] Singh R., Ichikawa T., Jain A., Awasthi K. (2021). 20—Zinc oxide as promising anode material for Li-ion battery. Nanostructured Zinc Oxide.

[14] Kim H., Jae W., Song J., Kim J. (2019). Skein-shaped ZnO/N-doped carbon microstructures as a high performance anode material for lithium-ion batteries. J. Alloys Compd..

[15] McCluskey M.D., Jokela S.J. (2009). Defects in ZnO. J. Appl. Phys..

[16] Pearton S.J., Norton D.P., Ip K., Heo Y.W., Steiner T. (2004). Recent advances in processing of ZnO. J. Vac. Sci. Technol. B Microelectron. Nanometer Struct. Process. Meas. Phenom..

[17] Klingshirn C., Hauschild R., Fallert J., Kalt H. (2007). Room-temperature stimulated emission of ZnO: Alternatives to excitonic lasing. Phys. Rev. B.

[18] Tang Z.K., Wong G.K.L., Yu P., Kawasaki M., Ohtomo A., Koinuma H., Segawa Y. (1998). Room-temperature ultraviolet laser emission from self-assembled ZnO microcrystallite thin films. Appl. Phys. Lett..

[19] Dong H., Zhou B., Li J., Zhan J., Zhang L. (2017). Ultraviolet lasing behavior in ZnO optical microcavities. J. Mater..

[20] Egerton E.J., Sood A.K., Singh R., Puri Y.R., Davis R.F., Pierce J., Look D.C., Steiner T. (2005). P-type doping utilizing nitrogen and Mn doping of ZnO using MOCVD for ultraviolet lasers and spintronic applications. J. Electron. Mater..

[21] Deng G., Zhang Y., Yu Y., Han X., Wang Y., Shi Z., Dong X., Zhang B., Du G., Liu Y. (2020). High-Performance Ultraviolet Light-Emitting Diodes Using n-ZnO/p-hBN/p-GaN Contact Heterojunctions. ACS Appl. Mater. Interfaces.

[22] Mohammad Reza M., Costas S. (2011). Metal Oxide ZnO-Based Varistor Ceramics. Advances in Ceramics.

[23] Levinson L., Philipp H. (1986). Zinc oxide varistors—A review. Am. Ceram. Soc. Bull..

[24] Raghu N., Kutty T.R.N. (1991). Varistors based on mixed-phase ceramics containing ZnO and negative temperature coefficient spinels. Appl. Phys. Lett..

[25] Park K., Lee J.K. (2007). Mn–Ni–Co–Cu–Zn–O NTC thermistors with high thermal stability for low resistance applications. Scr. Mater..

[26] Cho J., Hwang S., Ko D.-H., Chung S. (2019). Transparent ZnO Thin-Film Deposition by Spray Pyrolysis for High-Performance Metal-Oxide Field-Effect Transistors. Materials.

[27] Chen Z., Wang J., Wu H., Yang J., Wang Y., Zhang J., Bao Q., Wang M., Ma Z., Tress W. (2022). A Transparent Electrode Based on Solution-Processed ZnO for Organic Optoelectronic Devices. Nat. Commun..

[28] Burkov S.I., Zolotova O.P., Sorokin B.P., Turchin P.P., Talismanov V.S. (2018). Features of acoustic wave propagation in the Me/ZnO/Me/diamond waveguide structure. J. Acoust. Soc. Am..

[29] Shiosaki T., Kawabata A. (1974). Low-frequency piezoelectric-transducer applications of ZnO film. Appl. Phys. Lett..

[30] Galstyan V., Comini E., Baratto C., Faglia G., Sberveglieri G. (2015). Nanostructured ZnO chemical gas sensors. Ceram. Int..

[31] Hussain B., Ebong A., Ferguson I. (2015). Zinc oxide as an active n-layer and antireflection coating for silicon based heterojunction solar cell. Sol. Energy Mater. Sol. Cells.

[32] Vittal R., Ho K.-C. (2017). Zinc oxide based dye-sensitized solar cells: A review. Renew. Sustain. Energy Rev..

[33] Consonni V., Briscoe J., Kärber E., Li X., Cossuet T. (2019). ZnO nanowires for solar cells: A comprehensive review. Nanotechnology.

[34] Le A.T., Ahmadipour M., Pung S.-Y. (2020). A review on ZnO-based piezoelectric nanogenerators: Synthesis, characterization techniques, performance enhancement and applications. J. Alloys Compd..

[35] Pearton S.J., Ren F. (2014). Advances in ZnO-based materials for light emitting diodes. Curr. Opin. Chem. Eng..

[36] Žutić I., Fabian J., Das Sarma S. (2001). Spin injection through the depletion layer: A theory of spin-polarized p-n junctions and solar cells. Phys. Rev. B.

[37] Miccoli I., Spampinato R., Marzo F., Prete P., Lovergine N. (2014). DC-magnetron sputtering of ZnO: Al films on (00.1) Al_2_O_3_ substrates from slip-casting sintered ceramic targets. Appl. Surf. Sci..

[38] Liu B., Zeng H.C. (2003). Hydrothermal Synthesis of ZnO Nanorods in the Diameter Regime of 50 nm. J. Am. Chem. Soc..

[39] Schlur L., Calado J.R., Spitzer D. (2018). Synthesis of zinc oxide nanorods or nanotubes on one side of a microcantilever. R. Soc. Open Sci..

[40] Somvanshi D., Jit S. (2014). Analysis of Temperature-Dependent Electrical Characteristics of n-ZnO Nanowires (NWs)/p-Si Heterojunction Diodes. IEEE Trans. Nanotechnol..

[41] Wang Y., Li X., Wang N., Quan X., Chen Y. (2008). Controllable synthesis of ZnO nanoflowers and their morphology-dependent photocatalytic activities. Sep. Purif. Technol..

[42] Zhang N., Yi R., Shi R., Gao G., Chen G., Liu X. (2009). Novel rose-like ZnO nanoflowers synthesized by chemical vapor deposition. Mater. Lett..

[43] Meulenkamp E.A. (1998). Synthesis and growth of ZnO nanoparticles. J. Phys. Chem. B.

[44] Talam S., Karumuri S.R., Gunnam N. (2012). Synthesis, characterization, and spectroscopic properties of ZnO nanoparticles. Int. Sch. Res. Not..

[45] Hong R., Pan T., Qian J., Li H. (2006). Synthesis and surface modification of ZnO nanoparticles. Chem. Eng. J..

[46] Kattan P. (2011). Ratio of Surface Area to Volume in Nanotechnology and Nanoscience.

[47] Lu H., Liao L., Li J., Wang D., He H., Fu Q., Xu L., Tian Y. (2006). High Surface-to-Volume Ratio ZnO Microberets: Low Temperature Synthesis, Characterization, and Photoluminescence. J. Phys. Chem. B.

[48] Kumar H., Rani R. (2013). Structural and optical characterization of ZnO nanoparticles synthesized by microemulsion route. Int. Lett. Chem. Phys. Astron..

[49] Yıldırım Ö.A., Durucan C. (2010). Synthesis of zinc oxide nanoparticles elaborated by microemulsion method. J. Alloys Compd..

[50] Hasnidawani J., Azlina H., Norita H., Bonnia N., Ratim S., Ali E. (2016). Synthesis of ZnO nanostructures using sol-gel method. Procedia Chem..

[51] Vafaee M., Ghamsari M.S. (2007). Preparation and characterization of ZnO nanoparticles by a novel sol-gel route. Mater. Lett..

[52] Nagpal K., Rapenne L., Wragg D.S., Rauwel E., Rauwel P. (2022). The role of CNT in surface defect passivation and UV emission intensification of ZnO nanoparticles. Nanomater. Nanotechnol..

[53] Tsuzuki T., McCormick P.G. (2004). Mechanochemical synthesis of nanoparticles. J. Mater. Sci..

[54] Tsuzuki T., McCormick P.G. (2001). ZnO nanoparticles synthesised by mechanochemical processing. Scr. Mater..

[55] Tani T., Mädler L., Pratsinis S.E. (2002). Homogeneous ZnO nanoparticles by flame spray pyrolysis. J. Nanoparticle Res..

[56] Ghaffarian H.R., Saiedi M., Sayyadnejad M.A., Rashidi A.M. (2011). Synthesis of ZnO nanoparticles by spray pyrolysis method. Iran. J. Chem. Chem. Eng. IJCCE.

[57] Yang Y., Chen H., Zhao B., Bao X. (2004). Size control of ZnO nanoparticles via thermal decomposition of zinc acetate coated on organic additives. J. Cryst. Growth.

[58] Salavati-Niasari M., Davar F., Mazaheri M. (2008). Preparation of ZnO nanoparticles from [bis (acetylacetonato) zinc (II)]-oleylamine complex by thermal decomposition. Mater. Lett..

[59] Bekermann D., Gasparotto A., Barreca D., Maccato C., Comini E., Sada C., Sberveglieri G., Devi A., Fischer R. (2012). Co_3_O_4_/ZnO nanocomposites: From plasma synthesis to gas sensing applications. ACS Appl. Mater. Interfaces.

[60] Hiragino Y., Tanaka T., Takeuchi H., Takeuchi A., Lin J., Yoshida T., Fujita Y. (2016). Synthesis of nitrogen-doped ZnO nanoparticles by RF thermal plasma. Solid State Electron..

[61] Byrappa K., Ohara S., Adschiri T. (2008). Nanoparticles synthesis using supercritical fluid technology—Towards biomedical applications. Adv. Drug Deliv. Rev..

[62] Viswanathan R., Gupta R.B. (2003). Formation of zinc oxide nanoparticles in supercritical water. J. Supercrit. Fluids.

[63] Kinge S., Crego-Calama M., Reinhoudt D.N. (2008). Self-assembling nanoparticles at surfaces and interfaces. ChemPhysChem.

[64] Baruwati B., Kumar D.K., Manorama S.V. (2006). Hydrothermal synthesis of highly crystalline ZnO nanoparticles: A competitive sensor for LPG and EtOH. Sens. Actuators B Chem..

[65] Madathil A.N.P., Vanaja K., Jayaraj M. Synthesis of ZnO nanoparticles by hydrothermal method. Proceedings of the Nanophotonic Materials IV.

[66] Singh L., Sharma R., Singh N., Kumar A., Mahato D.K., Lee Y., Bechelany M., Mandal K.D. (2021). Semi-wet growth and characterization of multi-functional nano-engineered mixed metal oxides for industrial application. Prog. Cryst. Growth Charact. Mater..

[67] Huang M.H., Wu Y., Feick H., Tran N., Weber E., Yang P. (2001). Catalytic growth of zinc oxide nanowires by vapor transport. Adv. Mater..

[68] Prete P., Lovergine N., Tapfer L. (2007). Nanostructure size evolution during Au-catalysed growth by carbo-thermal evaporation of well-aligned ZnO nanowires on (100) Si. Appl. Phys. A.

[69] Hasanpoor M., Aliofkhazraei M., Delavari H. (2015). Microwave-assisted synthesis of zinc oxide nanoparticles. Procedia Mater. Sci..

[70] Bilecka I., Elser P., Niederberger M. (2009). Kinetic and thermodynamic aspects in the microwave-assisted synthesis of ZnO nanoparticles in benzyl alcohol. ACS Nano.

[71] Kahouli M., Barhoumi A., Bouzid A., Al-Hajry A., Guermazi S. (2015). Structural and optical properties of ZnO nanoparticles prepared by direct precipitation method. Superlattices Microstruct..

[72] Sharma R.K., Ghose R. (2015). Synthesis of zinc oxide nanoparticles by homogeneous precipitation method and its application in antifungal activity against *Candida albicans*. Ceram. Int..

[73] Lupan O., Emelchenko G.A., Ursaki V.V., Chai G., Redkin A.N., Gruzintsev A.N., Tiginyanu I.M., Chow L., Ono L.K., Roldan Cuenya B. (2010). Synthesis and characterization of ZnO nanowires for nanosensor applications. Mater. Res. Bull..

[74] Luther J.M., Gao J., Lloyd M.T., Semonin O.E., Beard M.C., Nozik A.J. (2010). Stability Assessment on a 3% Bilayer PbS/ZnO Quantum Dot Heterojunction Solar Cell. Adv. Mater..

[75] Choi J.J., Lim Y.-F., Santiago-Berrios M.E.B., Oh M., Hyun B.-R., Sun L., Bartnik A.C., Goedhart A., Malliaras G.G., Abruña H.D. (2009). PbSe Nanocrystal Excitonic Solar Cells. Nano Lett..

[76] Beek W.J., Wienk M.M., Kemerink M., Yang X., Janssen R.A. (2005). Hybrid zinc oxide conjugated polymer bulk heterojunction solar cells. J. Phys. Chem. B.

[77] Cullity B., Stock S. (2001). Elements of X-ray Diffraction.

[78] Bindu P., Thomas S. (2014). Estimation of lattice strain in ZnO nanoparticles: X-ray peak profile analysis. J. Theor. Appl. Phys..

[79] Saleem M., Fang L., Ruan H., Wu F., Huang Q., Xu C., Kong C. (2012). Effect of zinc acetate concentration on the structural and optical properties of ZnO thin films deposited by Sol-Gel method. Int. J. Phys. Sci.

[80] Ebin B., Arıg E., Özkal B., Gürmen S. (2012). Production and characterization of ZnO nanoparticles and porous particles by ultrasonic spray pyrolysis using a zinc nitrate precursor. Int. J. Miner. Metall. Mater..

[81] Koutu V., Shastri L., Malik M. (2016). Effect of NaOH concentration on optical properties of zinc oxide nanoparticles. Mater. Sci. Pol..

[82] Haque M.J., Bellah M.M., Hassan M.R., Rahman S. (2020). Synthesis of ZnO nanoparticles by two different methods & comparison of their structural, antibacterial, photocatalytic and optical properties. Nano Express.

[83] Mullin J.W. (2001). Crystallization.

[84] Habibi R., Daryan J.T., Rashidi A.M. (2009). Shape and size-controlled fabrication of ZnO nanostructures using novel templates. J. Exp. Nanosci..

[85] Montero-Muñoz M., Ramos-Ibarra J., Rodríguez-Páez J., Ramirez A., Huamaní-Coaquira J. Shape-control of Zinc Oxide nanoparticles: Enhancing photocatalytic activity under UV irradiation. Proceedings of the VIII International Congress of Engineering Physics.

[86] Jellison G., Modine F. (1996). Parameterization of the optical functions of amorphous materials in the interband region. Appl. Phys. Lett..

[87] Sapkota D.R. (2022). Characterization and Optimization of CuInSe2 Solar Cells Applicable for Tandem Devices. Ph.D. Thesis.

[88] Aspnes D., Theeten J., Hottier F. (1979). Investigation of effective-medium models of microscopic surface roughness by spectroscopic ellipsometry. Phys. Rev. B.

[89] Subedi I., Bhandari K.P., Ellingson R.J., Podraza N.J. (2016). Near infrared to ultraviolet optical properties of bulk single crystal and nanocrystal thin film iron pyrite. Nanotechnology.

[90] Oldham W. (1969). Numerical techniques for the analysis of lossy films. Surf. Sci..

[91] Lakhwani G., Roijmans R.F., Kronemeijer A.J., Gilot J., Janssen R.A., Meskers S.C. (2010). Probing charge carrier density in a layer of photodoped ZnO nanoparticles by spectroscopic ellipsometry. J. Phys. Chem. C.

[92] Yoshikawa H.Y.H., Adachi S.A.S. (1997). Optical constants of ZnO. Jpn. J. Appl. Phys..

[93] Malandrino G., Blandino M., Fragala M.E., Losurdo M., Bruno G. (2008). Relationship between nanostructure and optical properties of ZnO thin films. J. Phys. Chem. C.

[94] Zak A.K., Abrishami M.E., Majid W.H.A., Yousefi R., Hosseini S.M. (2011). Effects of annealing temperature on some structural and optical properties of ZnO nanoparticles prepared by a modified sol-gel combustion method. Ceram. Int..

[95] Zak A.K., Razali R., Majid W.H., Darroudi M. (2011). Synthesis and characterization of a narrow size distribution of zinc oxide nanoparticles. Int. J. Nanomed..

[96] Bian S.-W., Mudunkotuwa I.A., Rupasinghe T., Grassian V.H. (2011). Aggregation and Dissolution of 4 nm ZnO Nanoparticles in Aqueous Environments: Influence of pH, Ionic Strength, Size, and Adsorption of Humic Acid. Langmuir.

[97] Ha T.T., Canh T.D., Tuyen N.V. (2013). A Quick Process for Synthesis of ZnO Nanoparticles with the Aid of Microwave Irradiation. ISRN Nanotechnol..

[98] Kumbhakar P., Singh D., Tiwary C., Mitra A. (2008). Chemical synthesis and visible photoluminescence emission from monodispersed ZnO nanoparticles. Chem. Phys. Lett..

[99] Bindu P., Thomas S. (2017). Optical Properties of ZnO Nanoparticles Synthesised from a Polysaccharide and ZnCl_2_. Acta Phys. Pol. A.

[100] Chen J., Sun K.-W. (2010). Growth of vertically aligned ZnO nanorod arrays as antireflection layer on silicon solar cells. Sol. Energy Mater. Sol. Cells.

[101] Nam S.Y., Choi Y., Song Y.H., Jung M.H., Kang C.-M., Kong D.-J., Park S.J., Lee J.Y., Namkoong G., Lee D.-S. (2013). N-ZnO/i-InGaN/p-GaN heterostructure for solar cell application. Phys. Status Solidi A.

[102] Kaplan I. (1977). Nuclear Physics.

[103] Viezbicke B.D., Patel S., Davis B.E., Birnie D. (2015). Evaluation of the Tauc Method for Optical Absorption Edge Determination: ZnO Thin Films as a Model System. Phys. Status Solidi B.

[104] Varshni Y.P. (1967). Temperature dependence of the energy gap in semiconductors. Physica.

[105] Fan H. (1951). Temperature dependence of the energy gap in semiconductors. Phys. Rev..

[106] Manoogian A., Woolley J. (1984). Temperature dependence of the energy gap in semiconductors. Can. J. Phys..

[107] Zhang Y., Wang Z., Xi J., Yang J. (2020). Temperature-dependent band gaps in several semiconductors: From the role of electron–phonon renormalization. J. Phys. Condens. Matter.

[108] Wang M.-D., Zhu D.-Y., Liu Y., Zhang L., Zheng C.-X., He Z.-H., Chen D.-H., Wen L.-S. (2008). Determination of Thickness and Optical Constants of ZnO Thin Films Prepared by Filtered Cathode Vacuum Arc Deposition. Chin. Phys. Lett..

[109] Urbach F. (1953). The long-wavelength edge of photographic sensitivity and of the electronic absorption of solids. Phys. Rev..

[110] Kurik M.V. (1971). Urbach rule. Phys. Status Solidi A.

[111] Caponetti E. (2003). Synthesis, size control, and passivation of CdS nanoparticles in water/AOT/n-heptane microemulsions. Mater. Sci. Eng. C.

[112] Wang Y., Herron N. (1991). Nanometer-sized semiconductor clusters: Materials synthesis, quantum size effects, and photophysical properties. J. Phys. Chem..

