# Supplementary material for: Zinc Oxide Nanoparticles—Solution-Based Synthesis and Characterizations
Source: Nanomaterials (Basel). 2023 Jun 2;13(11):1795. doi: 10.3390/nano13111795 (PMC10255554; doi:10.3390/nano13111795)
Supplement: Supplementary file 1 [file nanomaterials-13-01795-s001.zip › nanomaterials-2410267-supplementary.pdf]

Zinc Oxide nanoparticles – solution-based synthesis and characterizations

Khagendra P. Bhandari[1], Dhurba R. Sapkota[2], Manoj K. Jamarkattel[2], Quenton Stillion[1],

Robert W. Collins[2]

[1]Department of Physics and Astronomy, Ohio Northern University, 525 S Main St. Ada OH 45810, USA.

[2]Wright Center for Photovoltaics, Innovation & Commercialization, University of Toledo, Toledo, OH 43606 USA.

Supplementary documents for ZnO synthesis

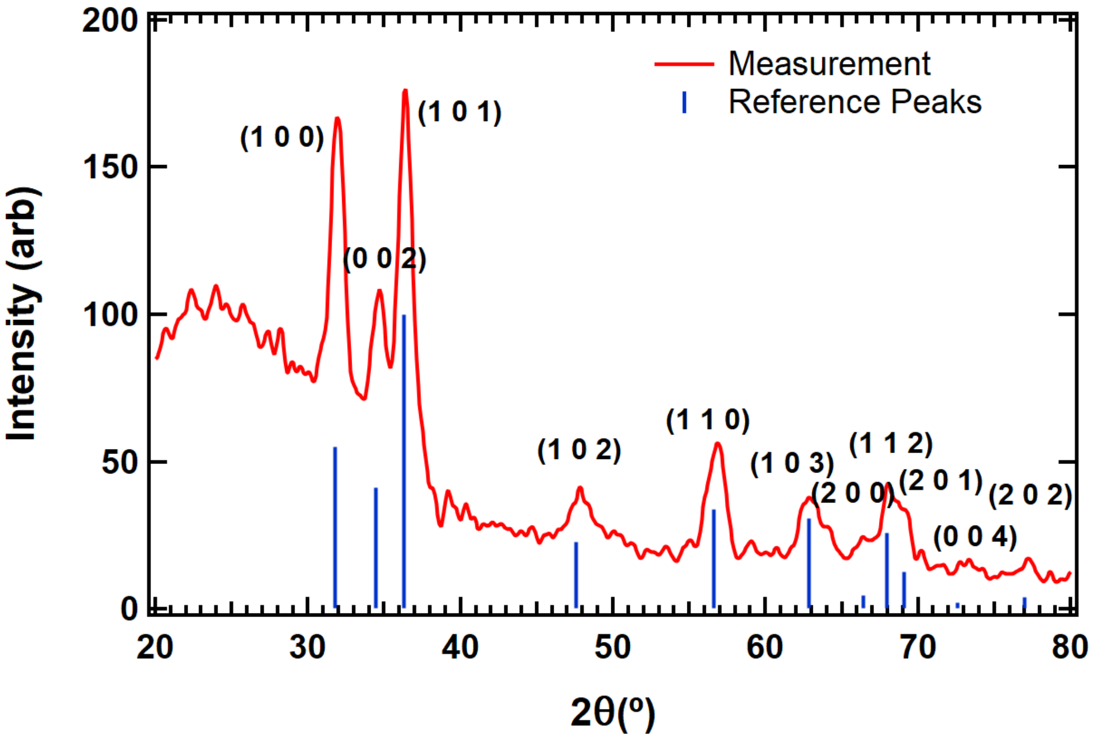

Figure S1. XRD patterns of as-synthesized ZnO thin film. The film was deposited by using spin coating process. The material is less crystalline because the peak intensities are comparatively lower.

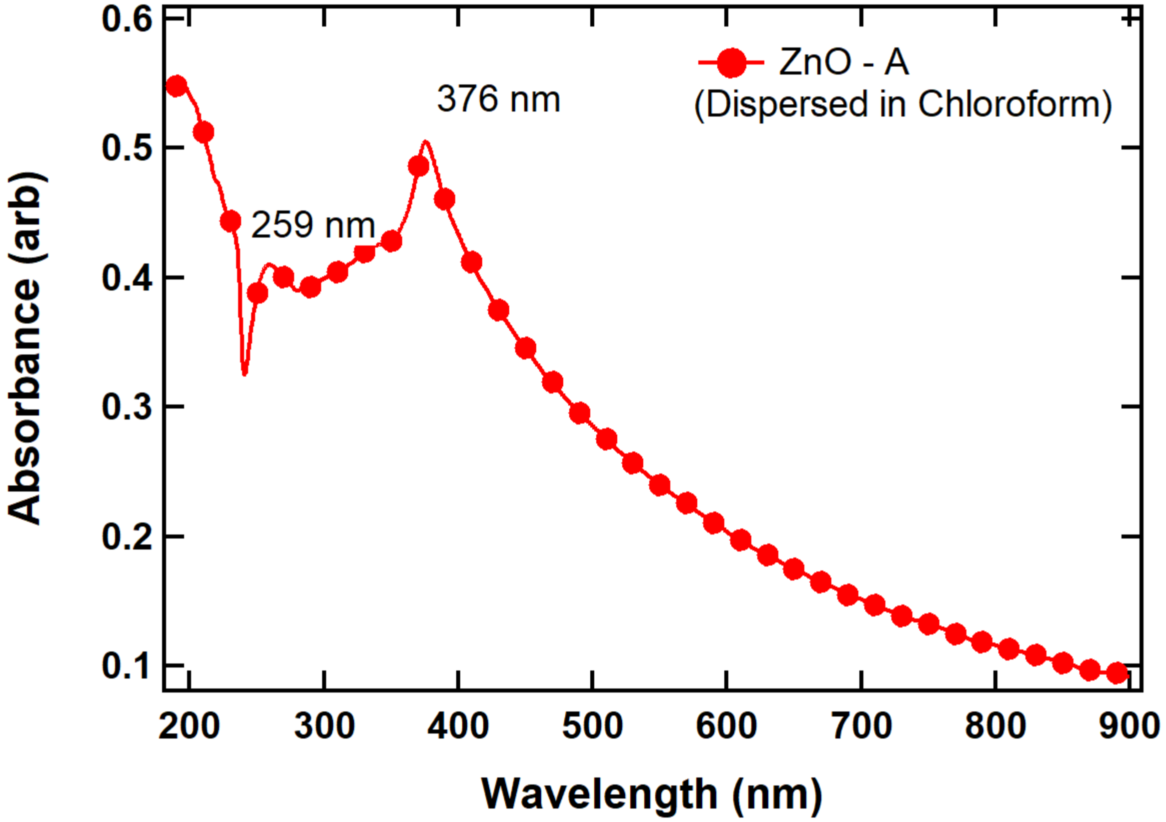

Figure S2. Absorbance spectrum of ZnO nanoparticles dispersed in chloroform. Synthesis was done by mixing all KOH solution in methanol at once into all $ZnAc_2$ solution in methanol.

The threshold wavelength can be calculated by analyzing UV/Vis spectrum according to the equation [1, 2].

$$\left(\frac{A}{\lambda}\right)^2 = K\left(\frac{1}{\lambda} - \frac{1}{\lambda_S}\right)$$

where, A, λ, and K are absorbance, wavelength, and an empirical constant respectively. If $\left(\frac{A}{\lambda}\right)^2$ versus $\frac{1}{\lambda}$ is plotted, above equation represents a straight line with y-intercept and the λ_S value can be obtained from the inter-section of the tangent drawn to inflection point with the baseline as shown. The threshold wavelength is 354.6 nm, only 20 nm higher than first exciton absorption peak.

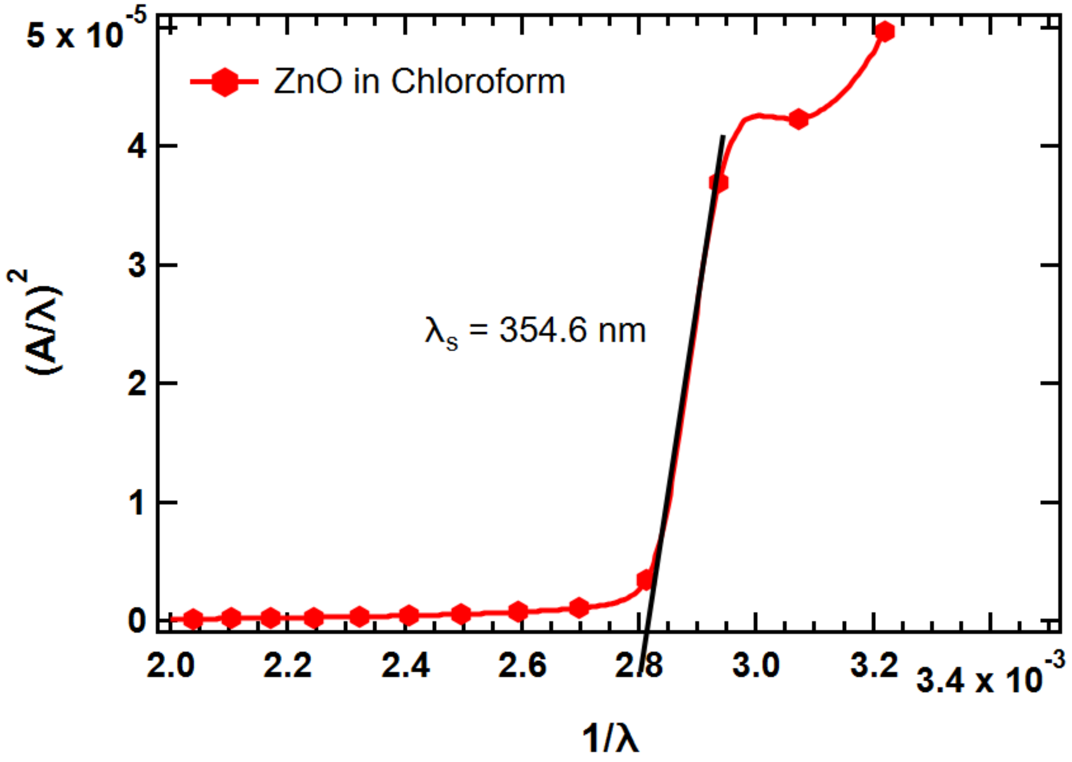

Figure S3. $(A/\lambda)^2$ vs. $1/\lambda$ of ZnO nanoparticles dispersed in chloroform.

1. Caponetti, E., et al., *Synthesis, size control, and passivation of CdS nanoparticles in water/AOT/n-heptane microemulsions.* Materials Science and Engineering: C, 2003. **23**(4): p. 531-539.
2. Wang, Y. and N. Herron, *Nanometer-sized semiconductor clusters: materials synthesis, quantum size effects, and photophysical properties.* The Journal of Physical Chemistry, 1991. **95**(2): p. 525-532.
